# Sarecycline interferes with tRNA accommodation and tethers mRNA to the 70S ribosome

**DOI:** 10.1073/pnas.2008671117

**Published:** 2020-08-12

**Authors:** Zahra Batool, Ivan B. Lomakin, Yury S. Polikanov, Christopher G. Bunick

**Affiliations:** ^a^Department of Biological Sciences, University of Illinois at Chicago, Chicago, IL 60607;; ^b^Department of Molecular Biophysics and Biochemistry, Yale University, New Haven, CT 06520;; ^c^Department of Dermatology, Yale University, New Haven, CT 06520;; ^d^Department of Pharmaceutical Sciences, University of Illinois at Chicago, Chicago, IL 60607;; ^e^Center for Biomolecular Sciences, University of Illinois at Chicago, Chicago, IL 60607

**Keywords:** sarecycline, tetracycline, antibiotic, 70S ribosome, X-ray structure

## Abstract

Sarecycline is a new narrow-spectrum tetracycline-class antibiotic approved for the treatment of acne vulgaris. Tetracyclines share a common four-ring naphthacene core and inhibit protein synthesis by interacting with the 70S bacterial ribosome. Sarecycline is distinguished chemically from other tetracyclines because it has a 7-[[methoxy(methyl)amino]methyl] group attached at the C7 position of ring D. To investigate the functional role of this C7 moiety, we determined the X-ray crystal structure of sarecycline bound to the *Thermus thermophilus* 70S ribosome. Our 2.8-Å resolution structure revealed that sarecycline binds at the canonical tetracycline binding site located in the decoding center of the small ribosomal subunit. Importantly, unlike other tetracyclines, the unique C7 extension of sarecycline extends into the messenger RNA (mRNA) channel to form a direct interaction with the A-site codon to possibly interfere with mRNA movement through the channel and/or disrupt A-site codon–anticodon interaction. Based on our biochemical studies, sarecycline appears to be a more potent initiation inhibitor compared to other tetracyclines, possibly due to drug interactions with the mRNA, thereby blocking accommodation of the first aminoacyl transfer RNA (tRNA) into the A site. Overall, our structural and biochemical findings rationalize the role of the unique C7 moiety of sarecycline in antibiotic action.

## Significance

Sarecycline is the first narrow-spectrum tetracycline-class antibiotic that was recently approved by the FDA for the clinical treatment of acne vulgaris. In this work, we determined two (2.8-Å and 3.0-Å) X-ray crystal structures of sarecycline bound to the initiation complex of the bacterial 70S ribosome and found that this antibiotic inhibits bacterial ribosome in part using a mechanism of direct mRNA contact, which has not been reported for any other tetracyclines so far. Moreover, our structural analysis rationalizes why sarecycline is able to overcome one of the most common mechanisms of resistance to tetracyclines among pathogenic bacteria. Thus, this work provides mechanistic insights into the function of the tetracycline class of antibiotics on the ribosome with direct clinical relevance.

**Fig. 1.**
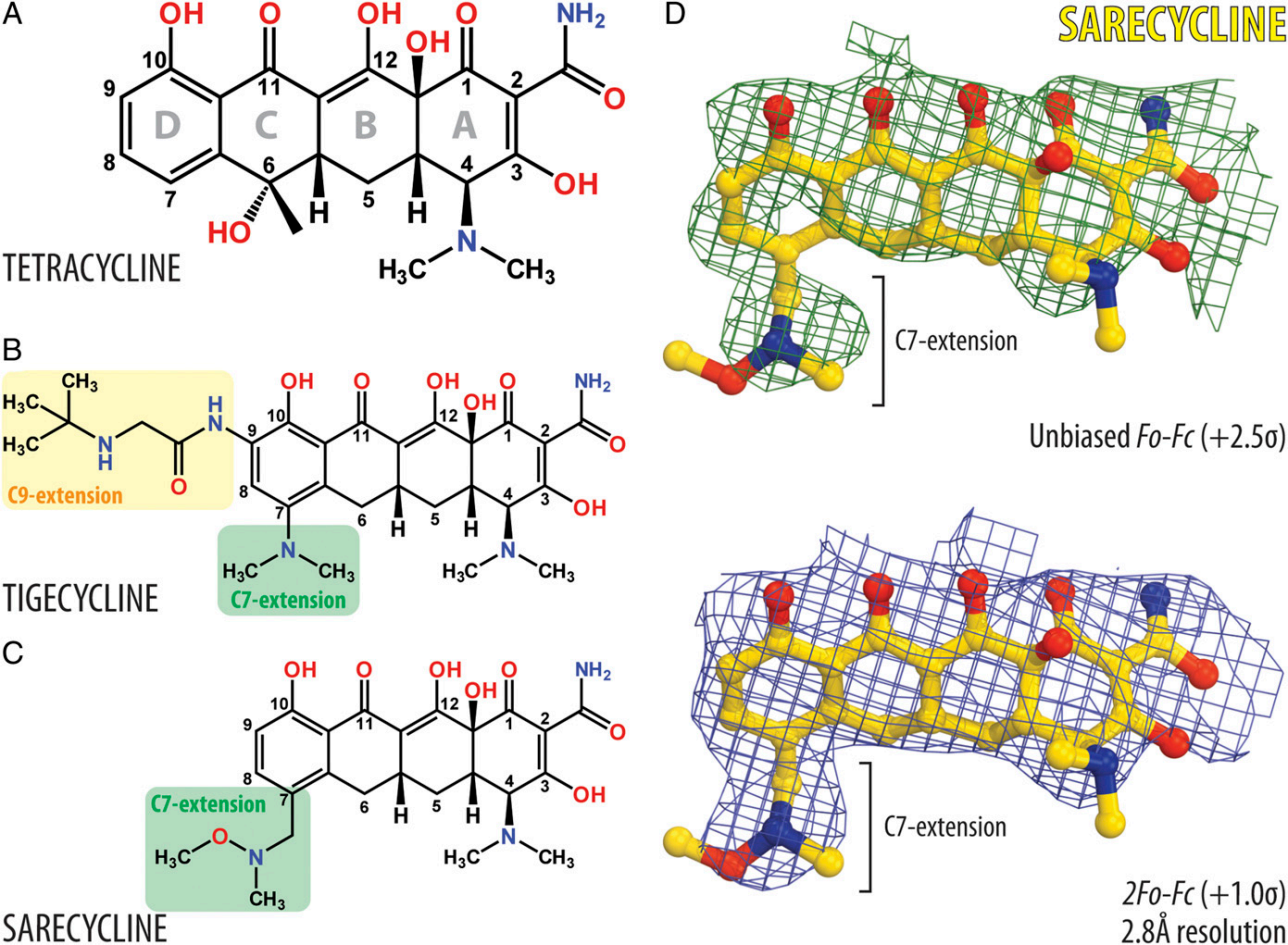
Chemical structures and electron density maps of ribosome-bound SAR. (*A*–*C*) Chemical structures of tetracycline-class antibiotics: TET (*A*), TIG (*B*), and SAR (*C*). The standard numbering of carbon atoms is indicated for each drug. Key chemical moieties are highlighted for TIG and SAR. (*D*) Unbiased *F*_o_*-F*_c_ (green mesh) and 2*F*_o_*-F*_c_ (blue mesh) electron difference Fourier maps of SAR in complex with the *T. thermophilus* 70S ribosome programmed with the UUC-mRNA. The refined model of SAR is displayed in the electron density maps contoured at 2.5 σ and 1.0 σ, respectively. Carbon atoms are colored yellow, nitrogens are blue, and oxygens are red.

Bacterial protein synthesis is targeted and inhibited by many small-molecule compounds (1). One such class of ribosome-targeting antibiotics is tetracyclines that share a common four-ring naphthacene core (Fig. 1*A*). Tetracyclines inhibit protein synthesis by binding at the A site of the small ribosomal subunit and blocking the accommodation of an incoming aminoacyl-transfer RNA (aa-tRNA) into the A site of the bacterial 70S ribosome (2–4). Single-molecule Förster resonance energy transfer (smFRET) data indicate that the presence of tetracyclines slows down the kinetics of aa-tRNA accommodation into the A site of the 70S ribosome (5). More specifically, tetracyclines bind at the decoding center (DC) between the head and the shoulder of the 30S subunit, in a pocket formed by helices 31 (h31) and 34 (h34) of the 16S ribosomal RNA (rRNA). This site partially overlaps with the anticodon stem loop (ASL) of the fully accommodated tRNA in the A site and, therefore, tetracyclines act by competing with tRNAs for binding to the ribosome. The polar edge of tetracycline also coordinates rRNA-bound magnesium ions, thereby forming additional interactions with the sugar-phosphate backbone of h31 and h34 in the head of the small ribosomal subunit, stabilizing tetracycline in the ribosome.

Tetracycline (TET, Fig. 1*A*) was the first prototype member of this antibiotic class and introduced into the market in 1947. Since then, several modified versions of it have been developed. TET derivatives have been prescribed for a variety of infections and inflammatory diseases, e.g., cholera, syphilis, and acne vulgaris (6). The observed broad-spectrum activity of tetracyclines can be explained by the seemingly common mode of binding and interaction of these drugs with the bacterial ribosome. Two particular tetracycline derivatives, minocycline and doxycycline, are readily prescribed by clinicians for the treatment of acne vulgaris and are the most used drugs today for the treatment of this disease. The success of tetracycline antibiotics in treating acne vulgaris has been attributed to their antimicrobial effects against *Cutibacterium acnes* (formerly known as *Propionibacterium acnes*) and antiinflammatory effects (such as the reduction of lipases) (7, 8). Both functional effects are thought to occur because tetracycline therapeutics inhibit protein synthesis by bacterial ribosomes.

Resistance to tetracyclines can be mediated either by the drug efflux pumps (9) or by the special ribosome protecting proteins (such as TetO/TetM) that resemble translation elongation factor G and induce conformational changes in the drug-binding pocket that prevent rebinding of the antibiotic (10, 11). Many of the tetracyclines that are in clinical use today carry chemical moieties that endow them with activity against tetracycline-resistant bacterial strains (12). For example, minocycline retains activity against bacteria-expressing *tet* genes, both encoding the tetracycline efflux pumps or ribosome-protection proteins. Members of another group of tetracycline derivatives, such as tigecycline (TIG, Fig. 1*B*), are active against common tetracycline-resistant bacterial strains due to their increased affinity to the ribosome binding site, which is mediated by the 9-t-butylglycylamido moiety attached to the ring D that forms a stacking interaction with the nucleotide C1054 of the 16S rRNA (13).

One of the recently developed tetracycline-class antibiotics that is also Food and Drug Administration-approved for the clinical treatment of acne vulgaris is sarecycline (SAR, Fig. 1*C*). This narrow-spectrum drug appears to have fewer undesirable side effects on the native human intestinal microflora, with ∼16- to 32-fold less activity against the gut microbiome, such as aerobic Gram-negative bacilli (14, 15). SAR is chemically distinguishable from other tetracycline antibiotics by the 7-[[methoxy(methyl)amino]methyl] group attached at the C7 position of the ring D (Fig. 1*C*) (16). This modification represents the longest and the largest C7 moiety among all of the tetracyclines and, therefore, it is especially curious whether the presence of such chemical groups could alter the mode of action of the drug.

In this work, we report the X-ray crystal structure of SAR in complex with the 70S ribosome from the Gram-negative bacterium *Thermus thermophilus* at 2.80-Å resolution. Although the overall binding site of SAR on the ribosome is the same as for other tetracyclines, our high-resolution structure allowed unambiguous placement of the SAR C7 moiety. It is evident from this structure that the C7 moiety of SAR directly interacts with the nucleotide at position (+6) of the A-site codon (position +6 relative to AUG codon and the translation start site), further stabilizing the drug in its binding pocket. This work provides experimental evidence suggesting that tetracyclines might interact with the messenger RNA (mRNA) on the ribosome and, despite sharing the same binding pocket, might exhibit quite different modes of action.

## Results and Discussion

### SAR Exhibits Idiosyncratic Activity In Vivo.

Differences in the chemical structure of SAR compared to TET prompted us to test whether mutations in the 16S rRNA that are known to confer resistance to TET can also confer resistance to SAR. To this end, we used a collection of previously obtained *Escherichia coli* strains, each of which is resistant to TET due to a single-nucleotide mutation in its 16S rRNA (Table 1 and *SI Appendix*, Fig. S1) (17). All of the TET-resistant strains were derived from the *E. coli* strains SQ110∆TolC and SQ171∆TolC, which, besides being hypersensitive to a large range of antibiotics due to the deletion of the gene for one of the major efflux pumps (Δ*tolC*), possess only one *rrn* operon encoding for the 16S and 23S rRNAs (18). All mutations were located in the vicinity of the decoding center on the small ribosomal subunit and immediately adjacent to the TET binding site. While most of the tested strains exhibited comparable resistance levels to both TET and SAR, one particular strain carrying U1060A mutation showed significantly higher resistance to SAR in comparison with TET (Table 1). Located in h34, nucleotide U1060 forms a base pair with the nucleotide A1197, which is in close proximity to the decoding center and immediately adjacent to the classical tetracycline binding site. Interestingly, mutation U1060A also confers strong resistance to a chemically unrelated drug, negamycin, which binds in the same site, but instead of competing with tRNA for binding to the ribosome interacts with the ASL of the incoming A-site tRNA and stabilizes its binding (*SI Appendix*, Fig. S2) (17). These data suggest that, although the binding sites of TET and SAR are largely overlapping, SAR could be more sensitive to certain changes in the overall geometry of the decoding center, indicating that some of the interactions of SAR with the ribosome might be different from those of TET.

**Table 1.** Minimal inhibitory concentrations (MICs) of TET and SAR against various *E. coli* strains carrying indicated mutations in the 16S rRNA

*E. coli* strain	Mutation	MIC, µg/mL (fold change relative to WT)	
		TET	SAR
SQ110∆TolC	WT	0.5	1.0
	16S: U1052G	0.125 (0.25)	0.5 (0.5)
SQ171∆TolC	WT	0.125	0.125
	G966U	0.5 (4)	2 (16)
	G1058C	0.5 (4)	1 (8)
	U1060A	0.5 (4)	4 (32)
	A1197U	2 (16)	2 (16)
	U1060A+A1197U	0.125 (1)	0.125 (1)

*E. coli* strain SQ110∆TolC carries only one *rrn* operon. *E. coli* strain SQ171∆TolC lacks all chromosomal *rrn* operons and, instead, carries pAM552 plasmid with a mutated rRNA operon.

### SAR Establishes Canonical Interactions with the 16S rRNA.

To understand the functional relevance of the C7 moiety of SAR (Fig. 1*C*) in the context of the translating ribosomal complex, we have determined the crystal structure of SAR bound to *T. thermophilus* 70S ribosome containing mRNA, and P-site–bound deacylated initiator tRNA_i_^Met^ at 2.80-Å resolution (*SI Appendix*, Table S1). In this experiment, we used mRNA containing phenylalanine UUC codon positioned in the A site of the ribosome. An unbiased difference Fourier map, calculated using the amplitudes from the crystals and phases derived from a model of the ribosome without the bound compound, revealed positive electron density resembling characteristic features of SAR (Fig. 1*D*). Although in our cocrystallization experiments we used higher concentrations of the drug (500 µM) than those used previously for structural studies of TET and its derivatives (4–80 μM), we observed SAR binding only at the primary tetracycline binding site and not at any of the secondary tetracycline binding sites reported earlier (2, 19). At 2.80-Å resolution, SAR can be unambiguously fitted into the obtained electron density map, including its C7 group (Fig. 1*D*).

**Fig. 2.**
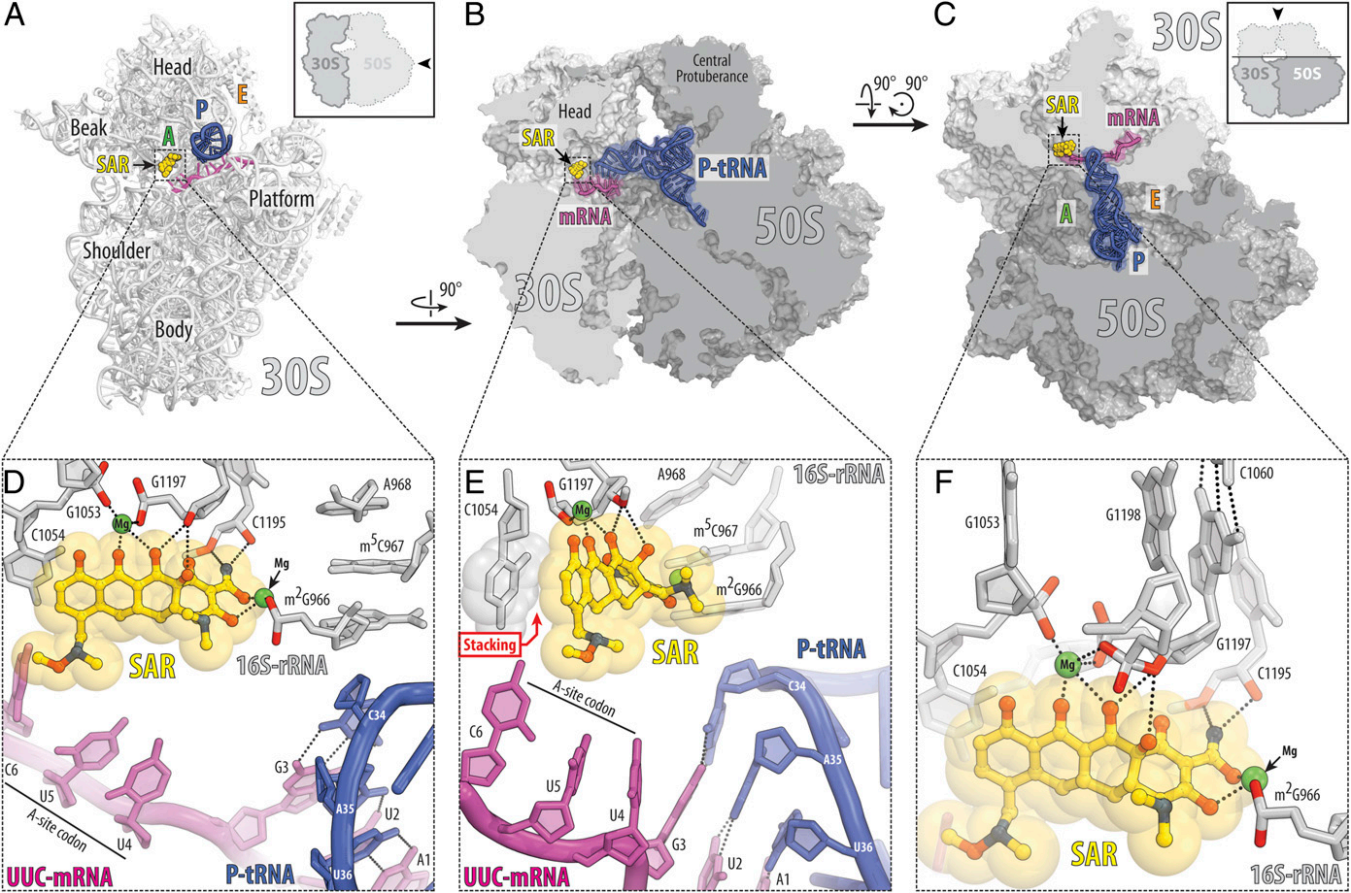
Structure of SAR in complex with the 70S ribosome, UUC-mRNA, and P-site tRNA at 2.8-Å resolution. (*A*–*C*) Overview of the SAR binding site (yellow) on the *T. thermophilus* 70S ribosome viewed from three different perspectives. The 30S subunit is shown in light gray, the 50S subunit is dark gray, the mRNA is magenta, and the P-site tRNA is colored dark blue. In *A*, the 30S subunit is viewed from the intersubunit interface, as indicated by *Inset* (the 50S subunit and parts of the P-site tRNA are removed for clarity). The view in *B* is a transverse section of the 70S ribosome. The view in *C* is from the top after removing the head of the 30S subunit and protuberances of the 50S subunit, as indicated by *Inset*. (*D*–*F*) Close-up views of the SAR interactions with the decoding center on the 30S ribosomal subunit. The *E. coli* numbering of the nucleotides in the 16S rRNA is used. Potential H bond interactions are indicated with dashed lines. Nucleotides of the mRNA are numbered relative to the first adenine in the P-site codon. Note that the C7 extension of SAR appears in close proximity to the third nucleotide of the A-site codon.

Our structure reveals binding of SAR in the canonical tetracycline site that is located in the A site on the small ribosomal subunit (Fig. 2 *A*–*C*). The overall binding pose of SAR is similar to that of TET and other tetracycline derivatives whose structures were reported previously (*SI Appendix*, Fig. S3) (2, 13). This is not surprising because the polar edge, which interacts with the 16S rRNA and is critical for drug binding, is the same between TET, TIG, and SAR. The polar edge of SAR directly contacts h31 and h34 of the 16S rRNA by forming hydrogen bonds (H-bonds) with the sugar-phosphate backbones of nucleotides C1195 and G1197 (Fig. 2 *D*–*F*). Similar to other tetracyclines, the polar edge of SAR also engages nucleotides G1053, G1197, and G1198 via a coordinated Mg^2+^ ion (Fig. 2 *D* and *F*) (13). Unlike TET, but similar to TIG, SAR also coordinates a second Mg^2+^ ion to enable an indirect interaction with the phosphate of the nucleotide m^2^G966 of h31 (Fig. 2 *D* and *F*).

**Fig. 3.**
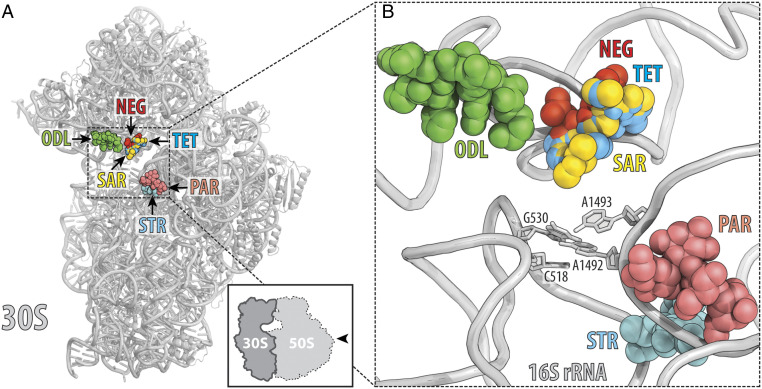
Antibiotics that bind in the decoding center on the small ribosomal subunit. (*A* and *B*) The location of the SAR binding site relative to the binding sites of other antibiotics known to target the decoding center of the small ribosomal subunit: TET (blue), negamycin (NEG, red), odilorhabdin NOSO-641 (ODL, green), streptomycin (STR, cyan), paromomycin (PAR, salmon). In *B*, the 16S rRNA nucleotides critical for decoding are shown as sticks.

Tetracyclines is not the only class of ribosome-targeting inhibitors that bind and act upon the decoding center (DC) of the bacterial ribosome. Distinct binding sites of the drugs usually indicate that the resistance mechanisms operating against one drug would be inefficient against the other. To check whether binding sites of SAR and other nontetracycline drugs overlap, we superimposed our structure of SAR in complex with the 70S ribosome with the known structures of other DC-binding inhibitors, such as negamycin, streptomycin, aminoglycoside antibiotic paromomycin, and peptide antibiotic odilorhabdin (Fig. 3). Our analysis shows a significant overlap only between the ribosome-bound SAR and negamycin but not with the other DC-binding drugs, suggesting that resistance mechanisms (involving ribosome) that are active against tetracyclines or negamycin, but not aminoglycosides or odilorhabdins, are also likely to be active against SAR.

### C7 Moiety of SAR Extends into the mRNA Channel and Interacts with the A-Site Codon.

In order to overcome various antibiotic resistance mechanisms, multiple synthetic tetracycline derivatives have been produced. Most of these compounds share the same polar edge, which is essential for drug binding to the bacterial ribosome, but incorporate various modifications at the opposite side of the molecule. For example, tigecycline (TIG, Fig. 1*B*) contains two extensions attached to the carbon atoms C7 and C9 of ring D. The enhanced potency of TIG can be rationalized by π–π stacking interactions between the C9 extension of TIG and the nucleotide C1054 of the 16S rRNA (13). Similar stacking interactions also exist for tetracyclines lacking the C9 extension, such as SAR (Fig. 2*E*) or TET (13). However, the degree of overlap of the stacking surfaces is noticeably smaller for these drugs. In contrast, the C7 group of SAR extends into the mRNA channel on the small ribosomal subunit, where it could potentially interact with the nucleotide +6 of the A-site codon in the mRNA (Fig. 2 *D* and *E*).

Structures of ribosome-bound tetracyclines typically do not contain tRNAs bound in the A site because these compounds compete with the incoming aa-tRNAs for binding to the A site on the 30S subunit. In turn, structures lacking an A-site tRNA, which stabilizes the A-site codon of the mRNA, also lack electron density for the mRNA in the A site. For example, in the previously reported structures of ribosome-bound TET or TIG, there is a complete absence of electron density for the mRNA in the A site (2, 13). Interestingly, despite the expected absence of a tRNA in the A site in our structure, we do observe continuous electron density corresponding to the A-site codon of the mRNA (*SI Appendix*, Fig. S4*A*), indicating that mRNA nucleotides must be stabilized by macromolecular interactions. Close examination of the SAR binding site revealed that the third nucleotide (cytidine) of the A-site UUC codon (position +6) is in close proximity to the C7 extension of SAR (Fig. 2 *D* and *E*). Although the oxygen of this C7 moiety could potentially act as an H bond acceptor, it is not within H bond distance from the exocyclic amino group of the cytidine residue in position +6 of the A-site codon in our structure (4.1 Å) (*SI Appendix*, Fig. S5). However, it is also evident from our structure that the methoxy group of the C7 extension of SAR forms a van der Waals contact with the cytidine in position +6 (Fig. 2 *D*–*F* and *SI Appendix*, Fig. S5), which is a weaker type of interaction compared to an H bond but still can provide additional stabilization of the mRNA, explaining why we observe strong electron density corresponding to the nucleotides of the A-site codon (*SI Appendix*, Fig. S4*A*). Curiously, when we modeled adenine in the position +6 of the A-site codon, the exocyclic N6 atom of such adenine residue appeared to be within H bond distance (3.0 Å) from the oxygen of the C7 moiety of SAR (*SI Appendix*, Fig. S5). This observation indicates that the C7 extension of SAR could potentially interact with the mRNA A-site codon, suggesting that SAR binding or its action could be affected by the mRNA context.

**Fig. 4.**
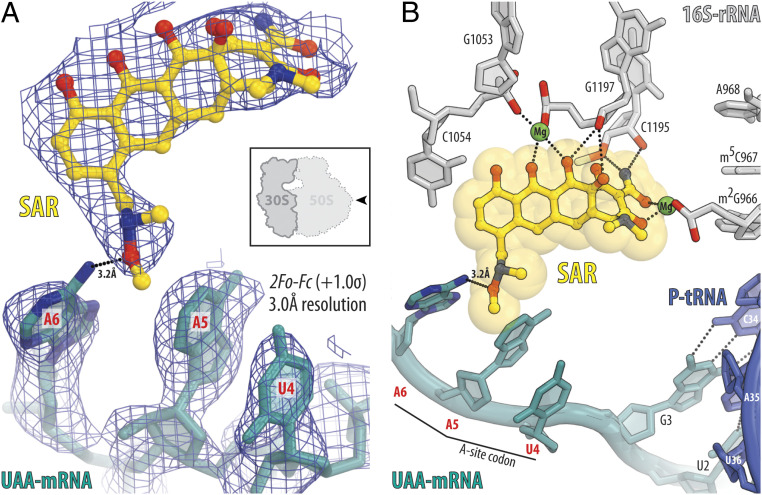
Structure of SAR in complex with the 70S ribosome, UAA-mRNA, and P-site tRNA at 3.0-Å resolution. (*A*) 2*F*_o_*-F*_c_ electron density map (blue mesh) of SAR (yellow) in complex with the *T. thermophilus* 70S ribosome programmed with UAA-mRNA (teal). The refined model of SAR and mRNA with UAA codon in the A site are displayed in the electron density map contoured at 1.0 σ. (*B*) Close-up view of the SAR interactions with the mRNA and the nucleotides of the decoding center of the 30S ribosomal subunit. The *E. coli* numbering of the nucleotides in the 16S rRNA is used. Note that the exocyclic amino group (N6 atom) of the third adenine residue in the codon is within H bond distance (3.2 Å) from the oxygen of the C7 moiety of SAR (dotted lines).

To verify whether SAR could directly interact with the mRNA on the ribosome, we determined an additional 3.0-Å resolution X-ray crystal structure of the 70S ribosome in complex with the P-site tRNA_i_^Met^, SAR, and an mRNA-containing adenine residue in the third position of the A-site codon (Fig. 4 and *SI Appendix*, Table S1). We observed electron density for the entire A-site codon, which was even stronger and more clear than for mRNA with the UUC codon (Fig. 4*A* and *SI Appendix*, Fig. S4*B*). Most importantly, the adenine residue in position +6 of the A-site codon forms H bond with the C7 extension of SAR (Fig. 4*B*), which is likely to stabilize mRNA, especially in the absence of the aa-tRNA in the A site. Altogether our structural data suggest that depending on the sequence of the A-site codon, SAR interacts not only with the 16S rRNA but also with the mRNA and interferes with the accommodation of the A-site tRNA by sterically hindering its access to the decoding center.

**Fig. 5.**
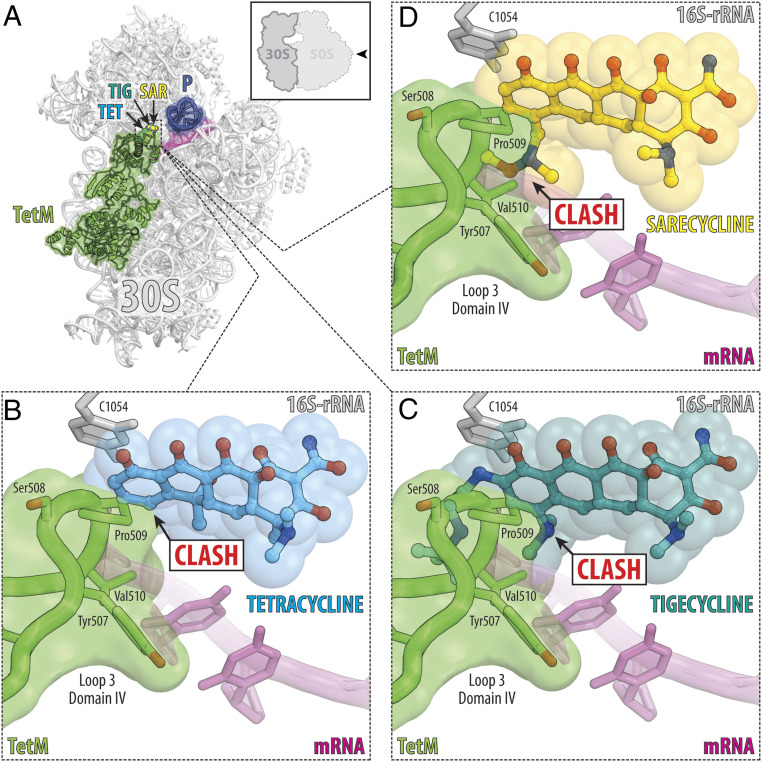
TET resistance involving ribosome protection protein TetM. (*A*) Superposition of the structure of ribosome-bound TetM protein (green, PDB ID code 3J9Y; ref. 21) with the structures of ribosome-bound TET (blue, PDB ID code 4V9A; ref. 13), TIG (teal, PDB ID code 4V9B; ref. 13), or SAR (yellow). All structures were aligned based on the 16S rRNA. mRNA is colored magenta; P-site tRNA is dark blue. (*B*–*D*) Close-up views of the sterical clashes between the amino acid residue(s) at the tip of the loop 3 of the domain IV of TetM and the ribosome-bound TET (*B*), TIG (*C*), or SAR (*D*). Note that TET exhibits clash only with the Pro509 residue, while the extended C9 and C7 moieties of TIG and SAR, respectively, exhibit more severe clashes with Ser508, Pro509, and Val510 residues of TetM.

Many bacteria acquire tetracycline resistance via ribosome protection proteins, such as TetM and TetO, which are paralogues of EF-G (9). These proteins bind to the A site of the tetracycline-stalled ribosome and expel tetracycline from its binding pocket (Fig. 5*A*) (3, 20). The structure of TetM bound to the *E. coli* 70S ribosome revealed that residue Pro509 at the tip of domain IV overlaps with the binding position of TET, explaining how TetM protein can dislodge ribosome-bound tetracycline molecule (Fig. 5*B*) (21). Interestingly, the binding of TIG to the ribosome is not affected by TetM, although a more severe clash between TetM and TIG was observed (Fig. 5*C*) (21). It has been proposed that increased affinity of TIG compared with TET, as well as the C9 extension of TIG hindering access of TetM to nucleotide C1054, together contribute to TIG’s ability to overcome TetM-mediated resistance (21). Similar to TIG, SAR is able to overcome the TetM-mediated resistance mechanism in vivo (16). Although SAR does not carry any C9 substituents (like TIG), the C7 extension can severely clash with the residues Pro509 and Val510 in the domain IV of TetM interfering with its ability to chase ribosome-bound SAR (Fig. 5*D*). Moreover, the observed additional contact of SAR with the mRNA on the ribosome is likely to result in SAR’s higher affinity compared to TET, which can also contribute to the reported ability of SAR to overcome TetM-mediated resistance and SAR’s low potential for inducing spontaneous mutations in Gram-positive bacteria (16).

### SAR Exhibits a Unique Mode of Action among Tetracyclines.

**Fig. 6.**
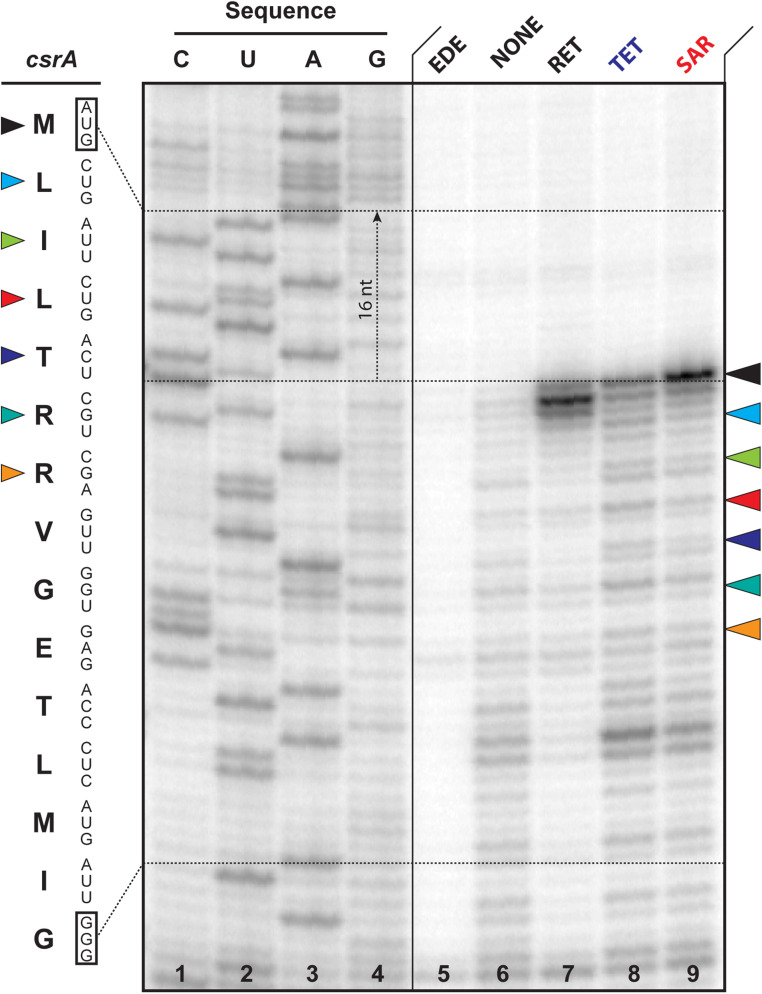
SAR inhibits the initiation step during protein synthesis. Ribosome stalling by SAR on *csrA* mRNA in comparison with other translation inhibitors (edeine, EDE; retapamulin, RET; and TET), as revealed by reverse-transcription primer-extension inhibition (toe-printing) assay in a cell-free translation system. *csrA* mRNA nucleotide sequence and the corresponding amino acid sequence are shown at *Left*. Colored triangles show ribosome stalling at various codons. Note that owing to the large size of the ribosome, the reverse transcriptase stops at the nucleotide +16 relative to the codon located in the P site.

To assess the mode of action of SAR and compare it to other tetracyclines, we used toe-printing analysis. This technique uses primer extension to detect antibiotic-induced ribosome stalling during in vitro translation of a model mRNA with a single-nucleotide precision (18, 22). This technique also allows for the determination of the context specificity of drug action (23). In general, tetracyclines inhibit the delivery of aa-tRNAs into the A site of the ribosome (*SI Appendix*, Fig. S2 *A* and *B*) and, therefore, act as elongation inhibitors (24–26). Because binding of the drug can occur at each elongation cycle, the resulting toe-printing pattern consists of multiple toe-printing bands separated by one codon (three nucleotides)—a pattern that is referred in the literature as ribosome stuttering (17, 18, 27). The addition of SAR to the cell-free transcription-translation system programmed with *csrA* mRNA resulted in dose-dependent ribosome stalling (*SI Appendix*, Fig. S6). Unlike TET, SAR causes the majority of ribosomes to stall at the initiator codon (Fig. 6, compare lanes 8 and 9). However, a small fraction of ribosomes manages to escape the start codon and continues translation, demonstrating a ribosome stuttering pattern similar to that of TET (Fig. 6, lanes 8 and 9). We observed this effect on different mRNA templates and at concentrations of the drug identical to those of TET. These data show that unlike TET, which interferes with the binding of aa-tRNA during each elongation cycle, SAR predominantly freezes ribosomes at the start codon during the initiation step of protein synthesis having a smaller effect on elongation. This is likely due to a higher affinity of SAR to the ribosome and the fact that SAR was added to the toe-printing mixture prior to the reaction onset. Moreover, this observation is consistent with our structural data showing that the C7 moiety of SAR extends into the mRNA channel, where it interacts with the nucleotide(s) of the A-site codon and stabilizes the mRNA.

Overall, our toe-printing experiments revealed that SAR stalls significantly more ribosomes at the start codon compared to TET. This is in contrast to the proposed previously general mechanism of action of tetracyclines that implies that these drugs can act equally well at each elongation cycle (13, 17, 26). Although most of the available in vitro biochemical data suggest that TET is an elongation inhibitor (26), there are reports from the in vivo studies showing that TET can be used to map translation start sites in the ribosome profiling experiments (28). Interestingly, our in vitro data with SAR corroborates previous in vivo data with TET. Alternatively, the strong ability of SAR to inhibit translation initiation and freeze ribosomes at the start codons can be rationalized by its higher affinity for the ribosome due to additional interactions with the mRNA in the A site. Such enhanced interaction of SAR with the ribosome is likely to block accommodation of the first incoming aa-tRNA to a greater extent than TET, which interacts only with the 16S rRNA. Apparently, the strong interaction of SAR with the ribosome can also occur during the translation elongation since SAR can potentially interact with other codons along the mRNA, making it a more potent translation inhibitor compared to TET.

To date, many of the studied ribosome-targeting antibiotics have been reported to exhibit a context-specific mode of action, which means that sequences of the mRNA or the peptides being synthesized by the ribosome influence the degree of inhibition by a drug (29). As a result, in the presence of a drug, the ribosome pauses at particular amino acid sequences more frequently than at others. The underlying mechanisms of context specificity of drug action are very different for different classes of drugs. SAR, with its unique C7 moiety extending into the mRNA channel where it could interact only with particular codons, is likely to exhibit mRNA sequence-dependent mode of action. Subsequent ribosome profiling experiments can provide additional insight into the context specificity of SAR action through the analysis of the transcriptome-wide distribution of ribosomes along the actively translated genes.

## Conclusions

We have determined the crystal structure of SAR bound to the bacterial ribosome initiation complex and identified that SAR, while binding to the same site in the small ribosomal subunit as other tetracyclines, establishes unique interaction by protruding its C7 moiety toward the mRNA binding channel and establishing interactions with the mRNA. The contact with mRNA might result in additional stabilization of the drug on the ribosome and an increased inhibitory effect of this antibiotic. Our study delineates both similarities and differences in the mechanism of ribosome binding and action between SAR and TET. These differences suggest SAR has its unique role in the tetracycline family, and clinicians should be aware of this as they evaluate the therapeutic potential for SAR. A structure-based approach toward understanding the mode of action of tetracycline derivatives can also inform medicinal chemists of the importance of such an approach for rational drug design.

## Materials and Methods

### Antibiotics Used for Biochemical and Structural Studies.

TET was purchased from Millipore Sigma. SAR was kindly provided by Allergan prior to Almirall purchasing the rights to SAR from Allergan in 2018.

### Determination of Minimal Inhibitory Concentrations.

All *E. coli* SQ110∆TolC strains carrying TET-resistance mutations in the 16S rRNA were grown in Luria-Bertani (LB) medium. Exponentially growing *E. coli* cells were diluted to a final culture density of OD_600_ = 0.001 and grown overnight at 37 °C in 96-well plates (100 µL per well) with increasing concentrations of antibiotics being tested, TET and SAR. The presence of live cells was determined by staining with AlamarBlue dye.

### Toe-Printing Assay.

The synthetic DNA template encoding the amino acid sequence MLILTRRVGETLMIGDEVTVTV (*SI Appendix*, Table S2) was initially generated by polymerase chain reaction (PCR) using *E. coli* BW25113 genomic DNA and AccuPrime Taq DNA Polymerase (Thermo Fisher Scientific). The sequences of the primers used for PCR are shown in *SI Appendix*, Table S3.

The toe-printing assay for drug-dependent translation stalling was performed as previously described in ref. 30 with minor changes. Toe-printing reactions were carried out in 5-µL aliquots containing PURExpress transcription-translation coupled system (New England Biolabs) with 0.5 picomoles of the DNA template added (27). The reactions were incubated at 37 °C for 15 min. Reverse transcription on the templates was carried out using radioactively labeled primer NV1 (*SI Appendix*, Table S3). Primer extension products were resolved on 6% sequencing gels as described previously (31). The final concentrations of SAR varied for different experiments.

### Crystallographic Structure Determination.

70S ribosomes from *T. thermophilus* (strain HB8) were prepared as described previously (32). Ribosome complexes with mRNA and tRNAs were formed as described previously (32). SAR was added to the preformed ribosome complexes to a final concentration of 250 µM prior to crystallization. All *Tth* 70S ribosome complexes were formed in the buffer containing 5 mM Hepes-KOH (pH 7.6), 50 mM KCl, 10 mM NH_4_Cl, and 10 mM Mg(CH_3_COO)_2_, and then crystallized in the buffer containing 100 mM Tris⋅HCl (pH 7.6), 2.9% (wt/vol) PEG-20K, 9–10% (vol/vol) 2-methyl-2,4-pentanediol (MPD), 175 mM arginine, 0.5 mM β-mercaptoethanol (33). Crystals were grown by the vapor diffusion method in sitting drops at 19 °C and stabilized as described previously (32), with SAR being added to the stabilization buffers (500 µM). Diffraction data were collected using beamlines 24ID-C and 24ID-E at the Advanced Photon Source (Argonne National Laboratory). A complete dataset for each ribosome complex was collected using 0.979 Å wavelength at 100 K from multiple regions of the same crystal using 0.3° oscillations (same as published previously; refs. 32 and 34). The raw data were integrated and scaled using the XDS software package (35). All crystals belonged to the primitive orthorhombic space group P2_1_2_1_2_1_ with approximate unit cell dimensions of 210 Å × 450 Å × 620 Å and contained two copies of the 70S ribosome per asymmetric unit. Each structure was solved by molecular replacement using PHASER from the CCP4 program suite (36). The search model was generated from the previously published structures of *T. thermophilus* 70S ribosome with bound mRNA and tRNAs (PDB ID code 4Y4P from ref. 32). The initial molecular replacement solutions were refined by rigid-body refinement with the ribosome split into multiple domains, followed by positional and individual B-factor refinement using PHENIX (37).

The atomic model of SAR was generated from its known chemical structure (Fig. 1*C*) using PRODRG online software (38), which was also used to generate restraints for subsequent energy minimization and refinement based on idealized three-dimensional geometry (similar to previously published procedure; ref. 39). Atomic models and restraints were used to fit/refine SAR into the obtained electron density map (Fig. 1*D*). The final model of *Tth* 70S ribosome in complex with mRNA/tRNAs and SAR was generated by multiple rounds of model building in COOT (40), followed by refinement in PHENIX (37). The statistics of data collection and refinement are compiled in *SI Appendix*, Table S1. All figures showing atomic models were generated using the PyMol software (https://pymol.org/2/).

## Supplementary Material

Supplementary File

## Data Availability

Coordinates and structure factors were deposited in the Research Collaboratory for Structural Bioinformatics Protein Data Bank with ID codes: 6XQD for the *T. thermophilus* 70S ribosome in complex with SAR, UUC-mRNA, and deacylated P-site tRNA_i_^Met^; and 6XQE for the *T. thermophilus* 70S ribosome in complex with SAR, UAA-mRNA, and deacylated P-site tRNA_i_^Met^.
