# Supplementary material for: Sarecycline interferes with tRNA accommodation and tethers mRNA to the 70S ribosome
Source: Proc Natl Acad Sci U S A. 2020 Aug 12;117(34):20530–7. doi: 10.1073/pnas.2008671117 (PMC7456112; doi:10.1073/pnas.2008671117)
Supplement: Supplementary File [file pnas.2008671117.sapp.pdf]

Supplementary Information Appendix for

Sarecycline interferes with tRNA accommodation and tethers mRNA to the 70S ribosome

Zahra Batool[1], Ivan B. Lomakin[2,3], Yury S. Polikanov[1,4,5,*], and Christopher G. Bunick[2,3,*]

[1] Department of Biological Sciences, University of Illinois at Chicago, Chicago, IL 60607, USA
[2] Department of Molecular Biophysics and Biochemistry, Yale University, New Haven, CT 06520, USA
[3] Department of Dermatology, Yale University, New Haven, CT 06520, USA
[4] Department of Pharmaceutical Sciences, University of Illinois at Chicago, Chicago, IL 60607, USA
[5] Center for Biomolecular Sciences, University of Illinois at Chicago, Chicago, IL 60607, USA

* To whom correspondence should be addressed:
E-mail: christopher.bunick@yale.edu (C.G.B.)
yuryp@uic.edu (Y.S.P.)

This file includes:

I. Supplementary Tables S1 to S3;

II. Supplementary Figures S1 to S6 with legends;

III. Supplementary References.

www.pnas.org/cgi/doi/10.1073/pnas.2008671117

I. SUPPLEMENTARY TABLES

Table S1. X-ray data collection and refinement statistics.

Crystals		**70S complex with P-site tRNA, SAR and UUC-mRNA**	**70S complex with P-site tRNA, SAR and UAA-mRNA**
Diffraction data			
Space Group		$P2_12_12_1$	$P2_12_12_1$
Unit Cell Dimensions, Å (a x b x c)		208.33 x 447.60 x 612.33	209.13 x 448.15 x 621.94
Wavelength, Å		0.9792	0.9792
Resolution range (outer shell), Å		224-2.80 (2.87-2.80)	311-3.00 (3.08-3.00)
$I/\sigma I$ (outer shell)		6.04 (0.84)	5.80 (0.86)
Resolution at which $I/\sigma I$=1, Å		2.80	3.00
Resolution at which $I/\sigma I$=2, Å		3.05	3.35
CC(1/2) at which $I/\sigma I$=1, %		13.1	10.0
CC(1/2) at which $I/\sigma I$=2, %		43.7	32.6
Completeness (outer shell), %		98.4 (99.4)	99.0 (99.2)
R_{merge} (outer shell)%		21.4 (166.2)	30.4 (177.8)
No. of crystals used		1	1
No. of Reflections Used:	Observed	5,485,119	4,917,037
	Unique	1,364,434	1,140,383
Redundancy (outer shell)		4.02 (4.06)	4.31 (4.40)
Wilson B-factor, Å^2		54.7	58.0
Refinement			
Resolution range of the diffraction data included in the refinement, Å		75-2.80	87-3.00
R_{work}/R_{free}, %		22.5/27.5	24.6/31.1
No. of Non-Hydrogen Atoms			
RNA		194,309	194,313
Protein		90,962	90,962
Ions (Mg, K, Zn, Fe)		2,727	2,668
Waters		3,424	2,882
Ramachandran Plot			
Favored regions, %		90.46	87.51
Allowed regions, %		8.26	10.92
Outliers, %		1.28	1.57
Deviations from ideal values (RMSD)			
Bond, Å		0.006	0.004
Angle, degrees		0.840	0.836
Chirality		0.042	0.042
Planarity		0.005	0.005
Dihedral, degrees		16.944	17.4
Average B-factor (overall), Å^2		59.1	54.9

Table S2. Nucleotide sequence of the *csrA* template used in the toe-printing experiments. Blue – T7 promoter; bold – open reading frame (ORF); red – annealing region of the toe-printing primer NV1.

Template	Nucleotide sequence (5'-to-3')
csrA	TAATACGACTCACTATAGGGATACAGAGAGACCCGACTCTTTTAATCTTTCAAGGAGCA AAGA**ATGCTGATTCTGACTCGTCGAGTTGGTGAGACCCTCATGATTGGGGATGAGGT CACCGTGACAGTTTAA**GTTAATAAGCAAAATTCATTATAACC

Table S3. DNA primers used for generating the templates for toe-printing analysis.

Primer name	Used for	Nucleotide sequence (5'-to-3')
T7-csrA-fwd	PCR	TAATACGACTCACTATAGGGATACAGAGAGACCCGA
csrA-rev-NV1	PCR	GGTTATAATGAATTTTGCTTATTAACTTAAACTGTCACGGTGACCTCATC
NV1	Toe-printing	GGTTATAATGAATTTTGCTTATTAAC

II. SUPPLEMENTARY FIGURES

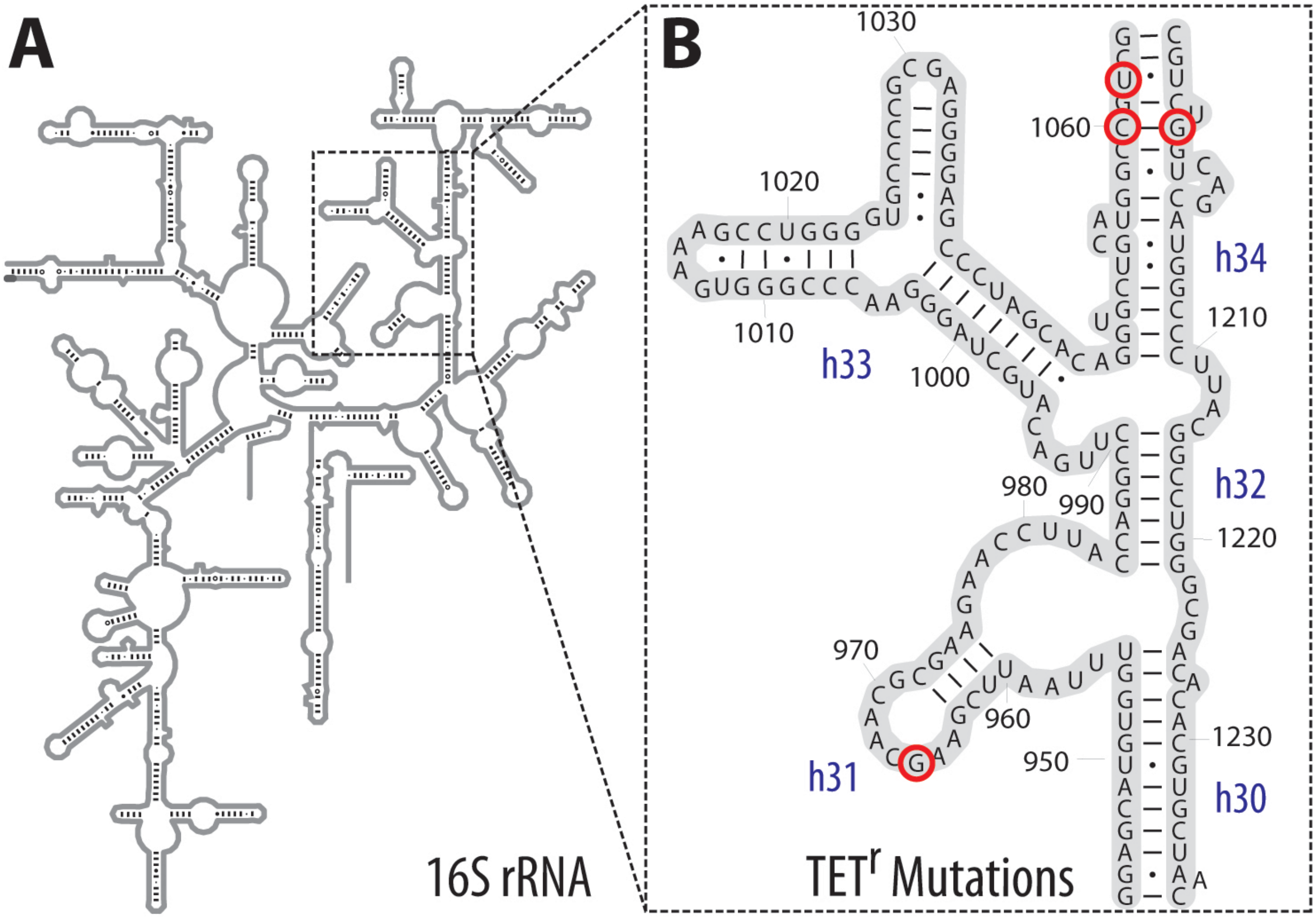

Fig. S1. Tetracycline-resistant mutations in the 16S rRNA. (**A**) The secondary structure of the entire 16S rRNA. (**B**) Close-up view of the secondary structure and the sequence of the 16S rRNA comprising helices 30-34. Positions of tetracycline-resistance mutations identified in *E. coli* are highlighted by red circles.

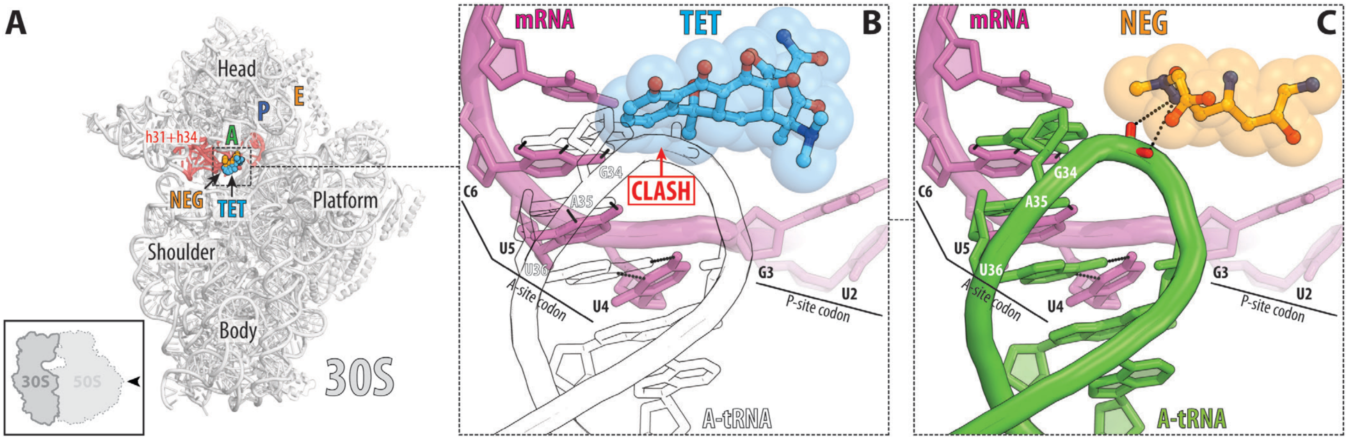

Fig. S2. Tetracycline and negamycin have overlapping binding sites but different mechanisms of action. (**A**) Overview of the superimposed binding sites of tetracycline (TET, blue) and negamycin (NEG, orange) in the 30S subunit. The view onto the 30S subunit is from the intersubunit interface, as indicated by the inset (the 50S subunit, mRNA and tRNAs are removed for clarity). Nucleotides constituting helices h31 and h34 of the 16S rRNA, which are involved in TET and NEG binding, are highlighted in pale red. The structure of ribosome-bound TET is from PDB entry 4V9A (1). The structure of ribosome-bound NEG is from PDB entry 4W2I (2). All structures were aligned based on h34 of the 16S rRNA. (**B**, **C**) Close-up views of the binding sites shown in (A). mRNA is shown in magenta, A-site tRNA is green. (**B**) Co-occupancy of the ribosome with TET and A-site tRNA is impossible because of the steric clash of the drug with the anticodon stem loop (ASL) of the incoming tRNA (shown as black contour to emphasize that it is absent from the structure). (**C**) Interaction of NEG with the ASL of the A-site tRNA stabilizes tRNA in the A site of the ribosome and results in tighter binding of tRNA to the A site.

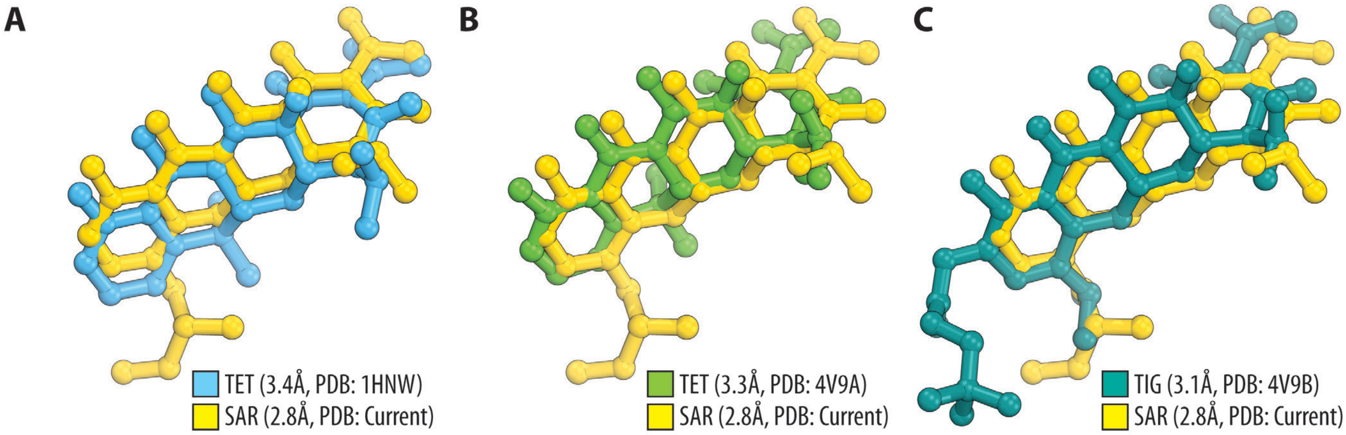

Fig. S3. Comparison of sarecycline (SAR) binding site with those of tetracycline (TET) and tigecycline (TIG). Superposition of the ribosome-bound SAR (yellow) and (**A**) TET (blue, PDB entry 1HNW (3)), (**B**) TET (green, PDB entry 4V9A (1)), or (**C**) TIG (teal, PDB entry 4V9B (1)). All structures of ribosome-bound tetracyclines were aligned based on h34 of the 16S rRNA.

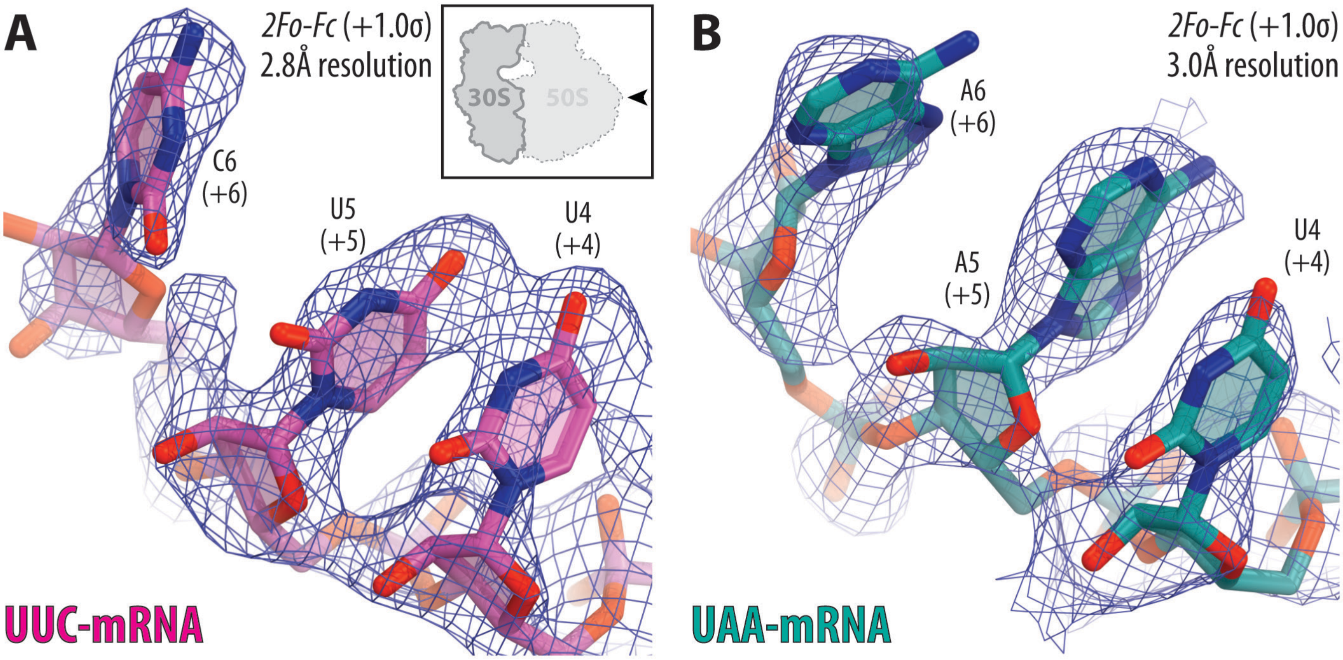

Fig. S4. Electron density maps of ribosome-bound mRNAs. (**A, B**) Observed $2F_o$-F_c electron density maps (blue mesh) corresponding to UUC (**A**, magenta) or UAA (**B**, teal) codons in the A site of the *T. thermophilus* 70S ribosome in complex with sarecycline and P-site tRNA. The refined models of ribosome-bound mRNAs are displayed in their respective electron density maps contoured at 1.0σ.

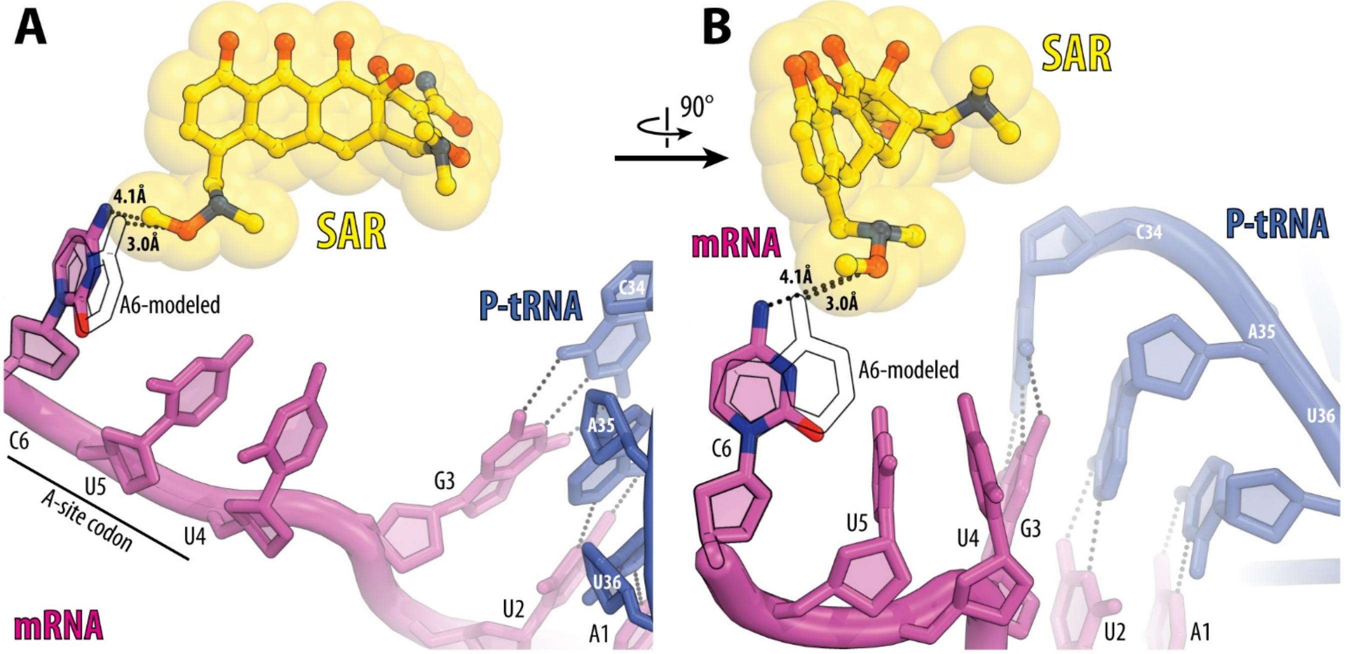

Fig. S5. *In silico* modeling of adenine in the third position of the A-site codon in the mRNA. Sarecycline (SAR) is colored yellow, mRNA is magenta, and P-site tRNA is dark blue. The third cytidine nucleotide (C6) of the A-site codon in the mRNA was mutated to adenine (A6-modeled). Note that the exocyclic amino group (N4 atom) of the cytidine residue is not within H-bond distance from the oxygen of the C7 moiety of SAR (4.1Å). In contrast, the exocyclic amino group (N6 atom) of the modeled adenine residue appears to be within the perfect H-bond distance (3.0Å) from the oxygen of the C7 moiety of SAR (dotted lines). The views in panels (A) and (B) are similar to the views in Figure 2D and 2E, respectively.

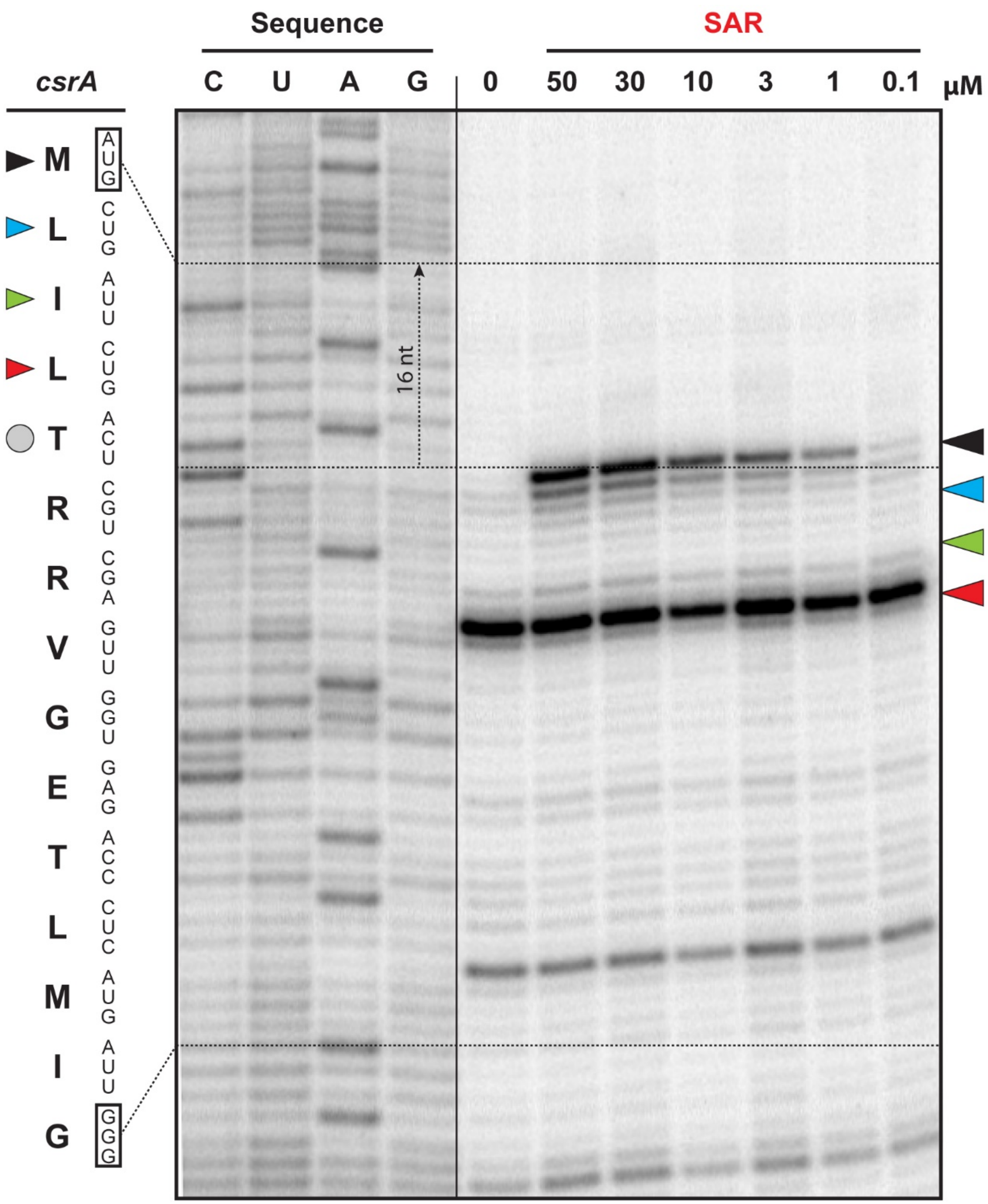

Fig. S6. Dose-dependent ribosome stalling on *csrA* mRNA in the presence of sarecycline. Toe-printing analysis of the ribosome stalling during translation of the *csrA* gene in the presence of increasing concentrations of sarecycline (SAR). mRNA nucleotide sequence and the corresponding amino acid sequence are shown on the left. Colored triangles indicate codon positions. The translation reactions were additionally supplemented with 50 µM of borrelidin, an inhibitor of threonyl-tRNA-synthetase, which results in depletion of the corresponding aminoacyl-tRNA and translation arrest at the 5th codon of the *csrA* open reading frame (marked with a grey circle) (4).

III. SUPPLEMENTARY REFERENCES

1. Jenner L*, et al.* (2013) Structural basis for potent inhibitory activity of the antibiotic tigecycline during protein synthesis. *Proc. Natl. Acad. Sci. USA* 110(10):3812-3816.

2. Polikanov YS*, et al.* (2014) Negamycin interferes with decoding and translocation by simultaneous interaction with rRNA and tRNA. *Mol. Cell* 56(4):541-550.

3. Brodersen DE*, et al.* (2000) The structural basis for the action of the antibiotics tetracycline, pactamycin, and hygromycin B on the 30S ribosomal subunit. *Cell* 103(7):1143-1154.

4. Vazquez-Laslop N*, et al.* (2011) Role of antibiotic ligand in nascent peptide-dependent ribosome stalling. *Proc. Natl. Acad. Sci. USA* 108(26):10496-10501.
